# Medication Underuse in Aging Outpatients with Cardiovascular Disease: Prevalence, Determinants, and Outcomes in a Prospective Cohort Study

**DOI:** 10.1371/journal.pone.0136339

**Published:** 2015-08-19

**Authors:** Andreas D. Meid, Renate Quinzler, Julia Freigofas, Kai-Uwe Saum, Ben Schöttker, Bernd Holleczek, Dirk Heider, Hans-Helmut König, Hermann Brenner, Walter E. Haefeli

**Affiliations:** 1 Department of Clinical Pharmacology and Pharmacoepidemiology, University of Heidelberg, Im Neuenheimer Feld 410, Heidelberg, Germany; 2 Division of Clinical Epidemiology and Aging Research, German Cancer Research Center (DKFZ), Heidelberg, Germany; 3 Saarland Cancer Registry, Saarbrücken, Germany; 4 Department of Health Economics and Health Services Research, Hamburg Center for Health Economics, University Medical Center Hamburg-Eppendorf, Germany; Texas Tech University Health Science Centers, UNITED STATES

## Abstract

**Background:**

Cardiovascular disease is a leading cause of death in older people, and the impact of being exposed or not exposed to preventive cardiovascular medicines is accordingly high. Underutilization of beneficial drugs is common, but prevalence estimates differ across settings, knowledge on predictors is limited, and clinical consequences are rarely investigated.

**Methods:**

Using data from a prospective population-based cohort study, we assessed the prevalence, determinants, and outcomes of medication underuse based on cardiovascular criteria from Screening Tool To Alert to Right Treatment (START).

**Results:**

Medication underuse was present in 69.1% of 1454 included participants (mean age 71.1 ± 6.1 years) and was significantly associated with frailty (odds ratio: 2.11 [95% confidence interval: 1.24–3.63]), body mass index (1.03 [1.01–1.07] per kg/m^2^), and inversely with the number of prescribed drugs (0.84 [0.79–0.88] per drug). Using this information for adjustment in a follow-up evaluation (mean follow-up time 2.24 years) on cardiovascular and competing outcomes, we found no association of medication underuse with cardiovascular events (fatal and non-fatal) (hazard ratio: 1.00 [0.65–1.56]), but observed a significant association of medication underuse with competing deaths from non-cardiovascular causes (2.52 [1.01–6.30]).

**Conclusion:**

Medication underuse was associated with frailty and adverse non-cardiovascular clinical outcomes. This may suggest that cardiovascular drugs were withheld because of serious co-morbidity or that concurrent illness can preclude benefit from cardiovascular prevention. In the latter case, adapted prescribing criteria should be developed and evaluated in those patients.

## Introduction

The predominant risks of a population with continuously increasing life expectancy include the simultaneous use of multiple medications [1], the associated risk of adverse drug reactions [2, 3], the growing complexity of medication schedules[4], and thus difficulty to follow the instructions and administer the drugs correctly (non-adherence) [5, 6]. However, the risk of not being prescribed all necessary drugs (underuse) is also common in aged populations [7] and increases with advancing age [8–11].

In cardiovascular disorders, underuse is frequent and well documented for many drug classes [7] including statins, antihypertensive agents, beta-blockers, antiplatelet agents, or drug combinations in the secondary prevention of cardiovascular events. Because those drugs that are not prescribed and not taken by the patient cannot be pharmacologically active, the prognosis of diseases is expectedly worse and particularly poor in patients with the highest risk for an event.

The **S**creening **T**ool to **A**lert doctors to **R**ight, i.e., appropriate, indicated **T**reatments (START) criteria is one of the preferred screening tools to detect underuse in older people [12]. Prevalence estimates of medication underuse defined by the START criteria differ across settings, and the current knowledge on factors influencing medication underuse defined by START criteria does not sufficiently consider the full heterogeneity of aging populations with regard to multiple morbidities, or frailty [8–11, 13–16]. Moreover, evidence for clinical consequences of medication underuse is scarce and methodological restraints preclude causality assessment, among them a cross-sectional study design [17], insufficient sample size in prospective investigations [18], or restrictions of the used data source [15]. This situation prompted us to address the topic in a prospective population-based cohort. We conducted a longitudinal analysis to examine the association of medication underuse with cardiovascular outcomes and determined the factors associated with medication underuse by applying precisely defined criteria for inclusion of participants with cardiovascular disease and determination of medication underuse. We hypothesized that appropriately treated participants would significantly differ from undertreated participants in terms of cardiovascular events and aimed to quantify the possible effect of medication underuse.

## Materials and Methods

### General Study Population

The study population is a subsample of the ESTHER study, a large population-based cohort study conducted in Germany described in detail elsewhere [19]. In brief, detailed participant information was collected from the participants and from their general practitioner (GP) by the use of standardized self-administered questionnaires at the baseline examination and four subsequent follow-ups after 2, 5, 8, and 11 years. Accordingly, self-reported diagnoses were validated with respect to occurrence and time. During the 8-year follow-up from July 2008 to December 2010, all eligible participants of the ESTHER cohort were asked to complete a 3-hour geriatric assessment conducted by trained study physicians at the participant’s home additionally including a brown-bag medication review.

In the home visit, 2714 participants provided medication records with at least one drug coded by the Anatomical Therapeutic Chemical (ATC) classification system. These participants were found eligible for the inclusion into our subsample. Those participants with a documented medical history of cardiovascular diseases according to adapted cardiovascular criteria from the START list from 2008 [12] were finally included (Table 1). Participants were followed up until the day they submitted their subsequent questionnaire or died. Thus, the day of the home visit with brown-bag medication review was considered their individual study entry.

**Table 1:** Adapted START criteria for determination of cardiovascular medication underuse.

criterion	description
A3	Antiplatelets with a documented history of atherosclerotic coronary, cerebral, or peripheral vascular disease^a^
A4	Antihypertensive therapy where systolic blood pressure consistently exceeded 160 mmHg in repeated measurements during the home visit
A5	Statin therapy with a documented history of coronary, cerebral, or peripheral vascular disease^a^ in a participant whose functional status indicated independence in activities of daily living as determined by a Barthel index score above 75
A8	Beta-blocker therapy in patients with chronic stable angina
F3	Antiplatelet therapy in diabetes mellitus with coexisting major cardiovascular risk factors^b^
F4	Statin therapy in diabetes mellitus if coexisting major cardiovascular risk factors are present^b^

^a^ a documented history of atherosclerotic coronary, cerebral, or peripheral vascular disease included previous myocardial infarction, stroke, coronary intervention (bypass surgery or balloon catheterization of the coronary arteries), pulmonary embolism, and deep vein thrombosis.

^b^ hypertension, hypercholesterolemia, and smoking history

### Data collection

The participants’ exposure to indicated medications was determined during a brown bag medication review, in which all drugs were unequivocally identified and recorded electronically by the study physician during the home visit. Information on drug utilization was complemented by the medical history of prevalent diseases at the time of the home visit in order to determine underuse according to the criteria listed in Table 1. Medications were identified by their ATC code (antiplatelet agents: B01AC; antihypertensives: C02, C03, C07, C08, or C09; beta-blockers: C07; statins: C10AA or C10BA).

We defined relevant study outcomes as cardiovascular mortality, non-fatal myocardial infarction, stroke, or coronary interventions such as bypass surgery or balloon catheterization of the coronary arteries. Non-cardiovascular mortality, i.e. death due to non-cardiovascular causes, was the competing event in competing risk analyses. We considered major changes in health status as concurrent events in a time-dependent covariate because they might provoke a reconsideration of the treatment or, more generally, might influence a participant’s covariate status. As such, we chose incident diagnoses of coronary heart disease, heart failure, renal failure, and pulmonary embolism.

Concerning covariates, co-morbidity was defined by the Cumulative Illness Rating Scale for Geriatrics (CIRS-G) as reported by the responsible GP. Information on income classes, presence of a partner or cohabitant, number of social contact persons, educational level (basic: ≤9, middle: 10–11, higher: ≥12 years), and smoking status (current, past, or never smoker) was extracted from the participant’s questionnaire. Physical examination during the geriatric assessment included two blood pressure measurements from both arms. The body mass index (BMI) was calculated based on weight and height values measured during the home visit. Calibrated devices were used to measure blood pressure, height, and weight. Validated assessment tools provided information on cognitive status (MMSE) and performance in activities of daily living (Barthel Index). We applied a modified phenotypic definition of frailty by Fried and co-workers [20] using population-independent cut-points for the physical frailty criteria grip strength, gait speed, and physical inactivity [21], whereas exhaustion and weight loss were obtained similarly to the original definition by Fried [20]. Participants meeting at least three of the criteria were considered “frail”, those with one or two criteria as “pre-frail”, and those without a positive criterion as “non-frail”. The visiting study physician also asked the participant about perceived information deficits on prescribed drugs (“do you feel sufficiently informed about how to take and apply your drugs”).

Vital status was ascertained via local population registries and information on the cause of death of fatal events were provided by public health departments providing the underlying cause of death according to the International Classification of Diseases, 10^th^ revision [ICD-10] with cardiovascular deaths being defined by ICD-10 codes I00–I99.

### Ethical approval

The study was approved by the Ethics Committees of the Medical Faculty of the University of Heidelberg (Study-ID: 058/2000) and of the Medical Board of the State of Saarland (Study-ID: 67/00) and was conducted in accordance with the declaration of Helsinki. Written informed consent was obtained from each participant prior to the study.

### Statistics

Standard descriptive methods were used to describe demographic characteristics of the study sample at baseline. Between-group differences were assessed using the Chi-square test for categorical variables and either the Student’s t-test or the Wilcoxon rank-sum test for ordinal or continuous variables, depending on their distribution.

Potential factors associated with medication underuse according to START criteria were determined in a logistic regression analysis. We applied variable selection by blocks: in the first block, variables with proven association (cognitive status and number of drugs [9–11]) were included together with variables considered as mandatory for adjustment (age, sex, and co-morbidity). In the second block, these variables were tested for inclusion by the backward elimination procedure. In the third block, forward selection was conducted to detect potential further predictors. We used Wald-tests with a selection limit of 10% (*P* = 0.1).

We conducted time-to-event analyses to study the potential impact of medication underuse on relevant cardiovascular events and competing deaths due to non-cardiovascular causes. Non-parametric methods using the log-rank test or the Gray test for cumulative incidence functions were applied in preliminary descriptive analyses. In the main analysis, we modeled cause-specific hazards while accounting for major concurrent health events as a time-dependent covariate. Besides mandatory covariates for adjustment (age, sex, co-morbidity, and presence of cardiovascular risk factors), we included variables with a significant association with the prescription status determined by the logistic regression model. The assessment of the proportional hazards assumption was checked and considered in the model building by means of Schoenfeld residuals. We further investigated sensitivity of our results by considering all-cause mortality as a part of our composite endpoint of relevant outcomes or applying multiple imputation of missing values.

All tests were two-tailed, 95% confidence intervals (CI) were calculated, and *P* values <0.05 were considered statistically significant. Statistical analyses were performed using the R software/environment version 3.0.2.

## Results

### Patient characteristics and prevalence of medication underuse

From the available 2714 medication records, 1454 (53.6%) participants fulfilled the inclusion criteria. The mean age (± standard deviation, SD) of included study participants was 71.1 ± 6.1 years (range 58–84) and 672 (46.2%) were female. Underuse defined as at least one missing medication was present in 1005 participants (69.1%). The demographic, clinical, medication-related, and socio-economic characteristics of the participants stratified for underuse or appropriate use revealed group differences at baseline for age, frailty, independence of daily life, and the number of drugs (Table 2).

**Table 2:** Baseline characteristics of the ESTHER subsample stratified for appropriate use and underuse according to START criteria.

Variables	Underuse		Appropriate use		All		
	N	value	N	value	N	value	Subgroupcomparison
**Sex: *frequencies (N) & column percentages (value in* %)**							
female	481	(47.9)	191	(42.5)	672	(46.2)	
male	524	(52.1)	258	(57.5)	782	(53.8)	*P*_Chi-square_ = 0.069
total	1005		449		1454		
**Age groups: *frequencies (N) & column percentages (value in* %)**							
<65	199	(19.8)	62	(13.8)	261	(18.0)	
65–74	565	(56.2)	273	(60.8)	838	(57.6)	
>75	241	(24.0)	114	(25.4)	355	(24.4)	*P*_Chi-square_ = 0.023
Mean ± SD		70.8 ± 6.2		71.7 ± 5.8		71.1 ± 6.1	*P*_t-test_ = 0.008
**Co-morbidity: CIRS-G-Score**							
Mean (Med)		8.5 (7)		8.8 (8)		8.5 (7)	*P*_Wilcoxon_ = 0.344
**Body mass index: BMI [kg/m** ^**2**^ **]**							
Mean ± SD		29.9 ± 5.2		29.4 ± 4.8		29.7 ± 5.1	*P*_t-test_ = 0.091
**Cognitive status: MMSE**							
Mean (Med)		27.9 (28)		28.1 (29)		27.9 (29)	*P*_Wilcoxon_ = 0.115
**Frailty categories: *frequencies (N) & column percentages (value in* %)**							
non-frail	250	(24.9)	131	(29.2)	381	(26.2)	
pre-frail	598	(59.5)	267	(59.5)	865	(59.5)	
frail	150	(14.9)	47	(10.5)	197	(13.6)	*P*_Chi-square_ = 0.034
**Independence of daily life: Barthel index**							
Mean (Med)		98.0 (100)		98.8 (100)		98.2 (100)	*P*_Wilcoxon_ = 0.020
**Smoking categories: *frequencies (N) & column percentages (value in* %)**							
current	73	(7.3)	38	(8.5)	111	(7.6)	
former	433	(43.1)	199	(44.3)	632	(43.5)	
non-smoker	483	(48.1)	201	(44.8)	684	(47.1)	*P*_Chi-square_ = 0.499
**Number of drugs: *frequencies (N) & column percentages (value in* %)**							
<5	407	(40.5)	100	(22.3)	507	(34.9)	
5–9	543	(54.0)	308	(68.6)	851	(58.5)	
>10	55	(5.5)	41	(9.1)	96	(6.6)	*P*_Chi-square_ <0.001
Mean (Med)		5.5 (5)		6.6 (6)		5.8 (6)	*P*_Wilcoxon_ <0.001
**Information deficit on drugs: *frequencies (N) & column percentages (value in* %)**							
N (%)	55	(5.5)	37	(8.2)	92	(6.3)	*P*_Chi-square_ = 0.059
**Income classes** ^a^ **: *frequencies (N) & column percentages (value in* %)**							
Mean (Med)		4.7 (5)		4.8 (5)		4.8 (5)	*P*_Wilcoxon_ = 0.392
**Number of reliable social contact persons**							
Mean (Med)		4.4 (4)		4.2 (3.5)		4.3 (4)	*P*_Wilcoxon_ = 0.362
**Education** ^b^ **: *frequencies (N) & column percentages (value in* %)**							
basic	695	(69.2)	297	(66.1)	992	(68.2)	
middle	155	(15.4)	76	(16.9)	231	(15.9)	
higher	134	(13.3)	73	(16.0)	206	(14.2)	*P*_Chi-square_ = 0.302

Med: Median; SD: standard deviation

^a^ Ordinal income classes per month were calculated as follows 1: less than 500 Euro; 2: 500 to 750 Euro; 3: 750 to 1000 Euro; 4: 1000 to 1500 Euro; 5: 1500 to 2000 Euro; 6: 2000 to 3000 Euro; 7: 3000 to 5000 Euro; 8: more than 5000 Euro

^b^ Classification: “basic” <9 years; “middle” 10–11 years; “higher” >12 years

In our subsample, more than two thirds of the participants had a documented history of atherosclerotic coronary, cerebral, or peripheral vascular disease. Among the 981 participants with a documented history of atherosclerotic disease, 50.9% did not use antiplatelet agents and 51.1% did not use a statin. Likewise, antiplatelet agents were missing in 50.4% and statins were withheld in 52.7% of the 546 participants with diabetes and at least one additional major cardiovascular risk factor. No beta-blocker was used by 31.3% of the 467 participants with chronic stable angina, and no antihypertensive drugs were used by 20.4% of the 270 participants with a systolic blood pressure above 160 mmHg.

### Predictors of medication underuse

To assess predictors of medication underuse in a multivariate way, we considered all variables listed in Table 2 in the specified variable selection procedure. While controlling for the mandatory covariates age (*P* = 0.106), sex (*P* = 0.024) and co-morbidity (*P* = 0.111) in the first block, both increasing number of drugs and impaired cognitive status were significant predictors (number of drugs *P*<0.001, MMSE *P* = 0.018). Subsequent stepwise forward selection included frailty (overall Wald test *P* = 0.012) and increasing BMI (*P* = 0.018).

**Fig 1:**
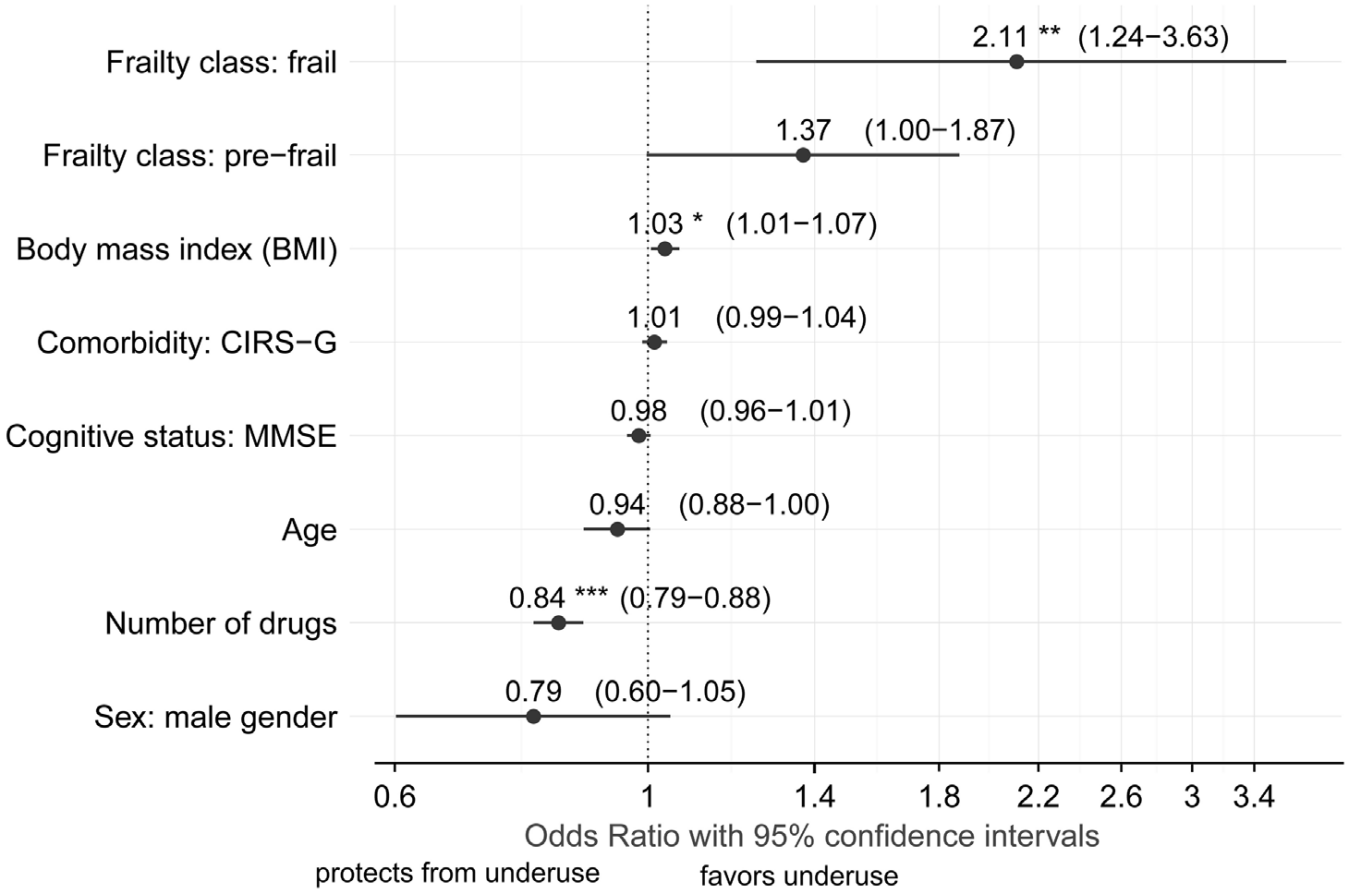
Presence of medication underuse was associated with frailty, BMI, and the number of drugs. Selected model variables and their association with medication underuse in a multivariate logistic regression model (*** < 0.001; ** < 0.01; and * < 0.05).

In the final model, statistical significance was observed for frailty, BMI, and the number of drugs (Fig 1A). Frail participants had more than two-fold increased odds of not receiving all indicated drugs (OR = 2.11, CI [1.24, 3.63], *P* = 0.006). BMI was likewise positively associated with the odds of not using an indicated drug (OR per kg/m^2^ = 1.03, CI [1.01, 1.07], *P* = 0.018) whereas the odds for underuse were reduced with increasing number of drugs (OR per drug = 0.84, CI [0.79, 0.88], *P*<0.001).

### Consequences of medication underuse

**Fig 2:**
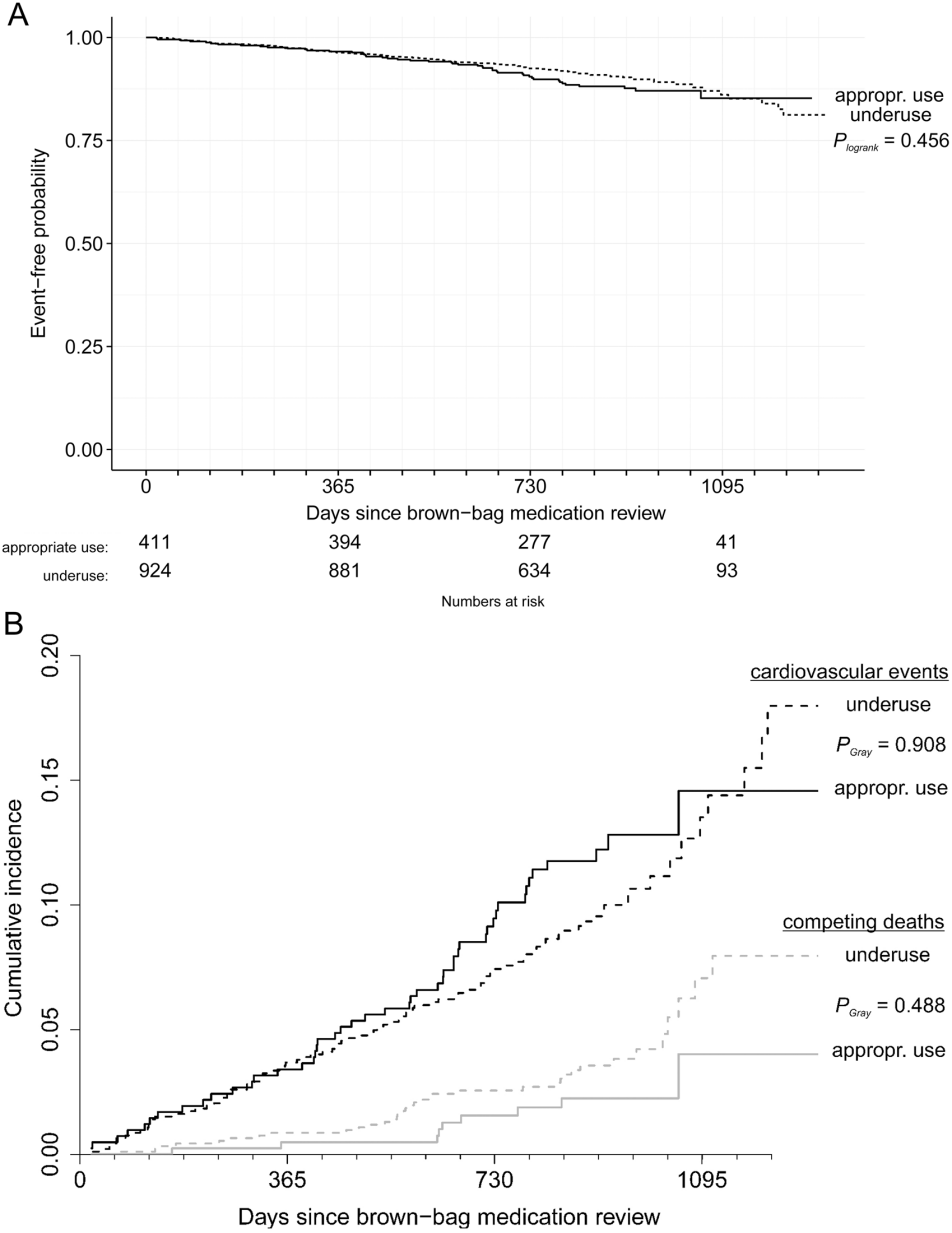
Medication underuse did not affect cardiovascular outcomes, but rather deaths due to non-cardiovascular causes. (A) Kaplan-Meier plot of relevant cardiovascular events for appropriate use and medication underuse (*P* value calculated by the log-rank test). (B) cumulative incidence functions of relevant (black) and competing events (gray) according to status of medication underuse (*P* value calculated by the Gray test) (solid line: appropriate use; dotted line: underuse).

1005 participants with medication underuse (77 lost to follow-up, 8.3%) and 449 appropriately treated participants (36 lost to follow-up, 8.7%) contributed a mean follow-up time of 2.24 years with a total number of 140 cardiovascular events. Neither a Kaplan-Meier plot (Fig 2A) (log rank test: *P* = 0.456) nor the corresponding cumulative incidence function (Fig 2B) (Gray test *P* = 0.908) revealed a difference in the composite endpoint of relevant cardiovascular events although results were more pronounced for competing deaths due to non-cardiovascular causes (Gray test *P* = 0.488).

In the multivariate regression model, medication underuse was not associated with cardiovascular outcomes (HR = 1.00, CI [0.65, 1.56], *P* = 0.99), but was significantly associated with non-cardiovascular deaths (HR = 2.52, CI [1.01, 6.30], *P* = 0.047) (Table 3). The parameters were consistent but attenuated in sensitivity analyses omitting the time-dependent covariate of concurrent health events or using multiple imputation, for which the assumption of missing at random was not deemed to be given. In sensitivity analyses including all-cause mortality into the relevant outcome, a trend to increased hazard ratios was observed (1.12 [0.87; 1.54]). The subgroup analysis with participants ≥65 years of age (N = 1193) showed more pronounced results: The hazard ratio for medication underuse regarding relevant cardiovascular events was raised to 1.17 (CI [0.72, 1.91], *P* = 0.516).

**Table 3:** Hazard ratios resulting from competing risk Cox regression analysis based on cause-specific hazards for relevant events (fatal and non-fatal cardiovascular events) and competing events (non-cardiovascular deaths) in the ESTHER cohort after brown bag medication review.

	Full Analysis Set						Subgroup ≥ 65 years					
Parameter^a^	Relevant events			Competing event			Relevant events			Competing event		
	HR	95% CI	*P*	HR	95% CI	*P*	HR	95% CI	*P*	HR	95% CI	*P*
Underuse	1.00	0.65, 1.56	0.987	2.52	1.01, 6.30	0.047	1.17	0.72, 1.91	0.516	3.63	1.23, 10.7	0.019
Health event	1.49	0.64, 3.43	0.354	^c^	^c^	^c^	1.80	0.78, 4.18	0.170	^c^	^c^	^c^
BMI [kg/m^2^]	0.96	0.91, 1.00	0.057	0.97	0.90, 1.04	0.372	0.95	0.90, 1.00	0.070	0.99	0.91, 1.07	0.734
Co-morbidity	1.05	1.02, 1.08	0.004	1.08	1.03, 1.13	0.002	1.06	1.02, 1.09	0.002	1.08	1.03, 1.13	0.001
CVD risk factor ^b^	2.79	0.68, 11.5	0.156	2.60	0.34, 19.9	0.358	2.54	0.61, 10.5	0.198	2.60	0.34, 20.1	0.360
Frailty	1.04	0.72, 1.50	0.838	1.75	0.96, 3.20	0.068	0.93	0.62, 1.38	0.708	1.69	0.90, 3.16	0.101

(BMI: body mass index; CI: confidence interval; CVD: cardiovascular disease; HR: hazard ratio)

^a^ Warranting the assumption of proportional hazards, the model was additionally stratified for sex, age groups, and categories of drug numbers as indicated by Schoenfeld residuals.

^b^ operationalized as a dichotomous variable indicating the presence of any cardiovascular risk factor (hypertension, hypercholesterolemia, and smoking history

^c^ no estimates are reported due to shortage of events leading to imprecise estimates with confidence intervals ranging to infinity

## Discussion

This subsample of the ESTHER study with a mean age of 71 years showed that medication underuse was common (69.1%), even though we enrolled only participants taking at least one drug. The prevalence is high compared to other studies [9, 11, 15, 16] likely reflecting that these earlier studies assessed rather different settings. The most frequently concerned use of statins is in line with previous findings reporting underuse in up to 60% of the participants [22, 23] and may generally reflect oversight, oblivion, ignorance, or controversial opinions among prescribing physicians [24, 25]. However, it might also stem from doubts about the therapeutic efficacy of certain drugs with inconclusive or missing reports for special populations such as frail older people.

With respect to factors potentially influencing medication underuse, we confirmed earlier reported predictors of underuse such as sex [8] and cognitive status [11]. In accordance with previous reports [16] and in contrast to others [9, 11], the likelihood of medication underuse declined with increasing age. In contrast to other studies [9, 11], we observed no association of underuse and co-morbidity, which may be attributed to different scales that were used to assess co-morbidity in earlier studies. If we assume that guidelines are implemented and no other influential factors are present, increasing the number of drugs would ultimately reduce the probability of being undertreated. Hence, while our result may appear expected and agrees with other studies [26], not all previous studies found an association with the number of drugs [1, 17] or even reported an increase [14] in the likelihood of underuse with increasing drug numbers. It is likely that patient characteristics related to functional status and physical disability will influence the prescribing behavior of physicians. As such, frailty is reportedly associated with reduced odds of receiving preventive medicines [27] and BMI is described to be inversely related to statin prescription [28]. Focusing on cardiovascular disease, we were the first to identify these indicators using explicit criteria for medication underuse.

We prospectively assessed the association of underutilization of preventive medications with cardiovascular outcomes. Only one study [15] systematically addressed this question in a similar way before. This study had a number of advantages over our study because its data source was larger (N = 4260) with a longer mean follow-up (4.5 years) and the population was older (mean age 77 ± 3.6 years). However, concurrently also several flaws may limit its findings (restriction to male gender, potential exposure misclassification due to non-validated self-reported medication history, calculation of a co-morbidity index based on records of hospital discharge diagnoses only, potential underestimation of events, and consideration of all-cause mortality). The authors observed a hazard ratio of 1.20 for the association with cardiovascular events after applying their own definitions of medication underuse. This result is similar to our naïve sensitivity analyses including all-cause mortality in the endpoint “cardiovascular events”. All-cause mortality can be misleading [29] and the findings of the previous study [15] might be biased upwards because it comprises a wide range of underlying causes of death.

The subgroup analysis of participants aged ≥65 years indicated an age-dependent effect of medication underuse. This presumed finding was sustained by the association detected for age as a continuous variable in the Cox regression model, clearly emphasizing that medication underuse becomes strikingly more important with increasing age: The hazard ratio of the undertreated population compared to the appropriately treated population increased by 7% with each year of life. This finding corroborates the age requirement ≥65 years as stated in the START criteria [12]. Nonetheless, guideline-recommended care, being the basis for START criteria, should be available to all patients including middle-aged patients [30].

In our population, medication underuse was unrelated to cardiovascular events of patients with cardiovascular disease, but was associated with a higher death rate from non-cardiovascular causes. A closer look at the actual death codes revealed that non-cardiovascular deaths were often reported as cancer. Although this does not imply that these patients ultimately died from cancer as opposed to cardiovascular causes, it might suggest a refusal to apply preventive cardiovascular medications in view of the participants’ co-morbidity. Frailty as an indicator of worse survival [20] was also weakly associated with non-cardiovascular events. Accordingly, remaining life expectancy, the expected (longer) time to benefit, and altered treatment preferences with higher age could have influenced patients and prescribers in the treatment choices.

Especially in everyday practice individual characteristics of a patient have to be considered, of which many would have led to the exclusion from the selected population of a randomized trial. Because (frail) older patients with a limited life expectancy are hardly ever included in those trials, our unexpected findings are especially valuable not only in the exploration of the preventive effectiveness of recommended medications, but also in the recognition of required further research with frail older patients who were often not adequately treated. The results raise the question of effectiveness in special populations such as frail older people.

The following potential limitations of our study design are worth to be considered. While the use of brown bag medication review adequately addressed the problem of underreporting, we had to extrapolate the stability of medication use over time and thus persistence and long-term exposure. Besides, further baseline variables may have changed over time. We took this issue into account by including major incident diagnoses as a time-dependent covariate reflecting the reason for potential changes in baseline variables such as the medication status. Finally, a larger sample size and a longer follow-up time would have been desirable. Considering our effect size of a literal null effect, no power recalculation was conducted.

## Conclusions

In conclusion, the thorough evaluation of actual medications and prevalent diagnoses in an aging ambulatory population with cardiovascular disease revealed that a sizeable fraction of this population met the criteria for underuse of cardiovascular drugs. Patients were less likely to use such medications if they were overweight, frail, and if they were prescribed less medicines. Medication underuse was not associated with increased risk for cardiovascular events but was associated with an increased risk for non-cardiovascular mortality. This may suggest that, at times, cardiovascular drugs are withheld in patients because of concurrent serious co-morbidity that potentially precludes benefit for pharmacological cardiovascular prevention. Future research is necessary to explore therapeutic effectiveness of preventive cardiovascular drugs in special populations such as frail older people.
